# Technology-assisted Total Hip Arthroplasty: A Contemporary Analysis of Regional Trends, National Trends, and 90-day Outcomes in a Nationwide Cohort

**DOI:** 10.1016/j.artd.2025.101949

**Published:** 2026-01-31

**Authors:** Amy Y. Zhao, Alex Gu, Gireesh Reddy, Bryant M. Song, Ilya Bendich, Andrew M. Schneider

**Affiliations:** a Department of Orthopaedic Surgery, The George Washington University School of Medicine and Health Sciences, Washington, DC, USA; b Department of Orthopaedic Surgery, Washington University in St. Louis School of Medicine, St. Louis, MO, USA

**Keywords:** Technology-assisted, Computer navigation, Robotic, Total hip arthroplasty, Trends, Outcomes

## Abstract

**Background:**

Technology-assisted total hip arthroplasty (THA)—including computer-navigated and robotic-assisted techniques—has emerged as a strategy to enhance component alignment and potentially improve postoperative outcomes. Although prior studies have described increasing utilization, contemporary trends and associated complication rates remain underexplored.

**Methods:**

A retrospective cohort study was conducted using a large national database to identify patients who underwent primary elective THA between 2010 and 2023. Patients were stratified into conventional vs technology-assisted THA groups, with the latter defined by the use of computer navigation or robotic assistance. Annual utilization trends were evaluated using linear regression, and 90-day postoperative complications were compared using multivariate logistic regression after adjusting for demographic, clinical, and regional factors.

**Results:**

Among 1,062,597 patients undergoing primary elective THA, 4% received technology-assisted procedures. Utilization increased from 1.2% in 2010 to 12% in 2023—a 927% relative increase. Regional variation was notable, with highest utilization in the Northeast and the lowest in the Midwest. Technology-assisted THA was associated with lower odds of 90-day complications (5.36% vs 6.26%; adjusted odds ratio [OR]: 0.77; 95% confidence interval [CI]: 0.75-0.80), particularly reduced odds of dislocation (OR: 0.64; 95% CI: 0.60-0.69) and periprosthetic joint infection, though with higher odds of wound dehiscence (OR: 1.15; 95% CI: 1.07-1.23).

**Conclusions:**

Utilization of technology-assisted THA has increased substantially across the United States, accompanied by improved short-term outcomes, most notably decreased dislocation. These findings support the potential clinical benefits of surgical technology in THA, while underscoring the need for ongoing evaluation of long-term results.

## Introduction

Total hip arthroplasty (THA) is a widely successful and cost-effective intervention for patients with end-stage osteoarthritis, offering substantial improvements in pain, mobility, and quality of life [1,2]. However, postoperative complications such as dislocation, infection, and periprosthetic fracture (PPF) remain key contributors to morbidity and revision surgery [3,4].

In response, technology-assisted THA, including computer navigation and robotic assistance, has emerged as a strategy to improve component alignment and potentially improve postoperative outcomes [5,6]. Recent studies have documented increasing adoption of technology-assisted THA. Hsiue et al. found that technology-assisted procedures rose significantly from 2005 to 2014 [7]. Similarly, Korber et al. reported that the national utilization of computer-navigated THA and robotic-assisted THA has grown significantly from 2005 to 2018 [8]. However, there is a paucity of literature assessing more recent trends in utilization, particularly in the context of recent technological advancements and the adoption of new robotic-assistance platforms for THA.

In parallel, it remains unclear whether technology-assisted THA results in improved clinical outcomes. While proponents argue that enhanced accuracy in component positioning may reduce the risk of dislocation, revision, and other complications, the evidence is mixed [9]. For instance, Kunze et al. found no significant difference in all-cause revision between robotic-assisted and conventional THA, while Piple et al. reported increased rates of PPF associated with robotic-assisted procedures [10,11]. These conflicting findings underscore the need for further investigation, particularly in light of evolving technologies.

Given the increasing use of robotic- and computer-assisted platforms in THA and the ongoing debate regarding their clinical utility, this study aims to (1) evaluate national trends in the utilization of technology-assisted THA from 2010 to 2023 and (2) compare 90-day postoperative outcomes between technology-assisted and conventional THA. We hypothesized that recent innovations have translated into broader adoption and improved short-term outcomes following primary THA.

## Material and methods

A retrospective cohort study was conducted using the PearlDiver Administrative Claims Database (PearlDiver Technologies Inc., Colorado Springs, CO). The Mariner dataset was utilized, consisting of all-payer claims information for over 170 million patients across the United States. All procedures and complications were identified using Current Procedural Terminology (CPT) and International Classification of Diseases (ICD) codes Ninth and Tenth Edition. As all patient information was deidentified in the dataset, institutional review board approval was not needed.

Patients who underwent primary elective THA were identified using CPT, ICD-9, and ICD-10 codes (Appendix 1). Patients undergoing THA for nonelective indications, including fracture and malignancy, who had less than 90 days of claims records or who underwent bilateral THA were excluded. Patients were then stratified into 2 groups, conventional THA and technology-assisted THA. For this study, technology-assisted THA was defined as primary THA performed using either computer navigation or robotic systems, as identified by relevant CPT and ICD procedural codes. Cases without navigation or robotic assistance were categorized as conventional THA (Appendix 1). Demographic and clinical characteristics were observed for all patients, including age, sex, Elixhauser Comorbidity Index, Charlson Comorbidity Index, and payer type (cash, commercial, government, Medicare, Medicaid, unknown) [12,13]. Ninety-day complications included acute myocardial infarction, mechanical complications, pulmonary embolism, pneumonia, sepsis, wound dehiscence, periprosthetic joint infection (PJI), PPF, and dislocation. Mechanical complications included prosthesis-related mechanical issues such as mechanical loosening, implant failure, instability, and other device-related complications, as defined by the ICD-9 and ICD-10 codes listed in Appendix 1. All-cause complications requiring readmission at 90 days were also observed.

Continuous variables were presented as means (standard deviation) and compared using Student’s *t*-test. Categorical variables were presented as frequencies and percentages and compared using chi-square test. Multivariate logistic regression was used to determine risk of 90-day complications after adjusting for patient characteristics, including demographics, comorbidities, regionality, insurance type, and technology assistance. To determine annual trends, Spearman’s correlation and linear regression were used. A *P* value below 0.05 was defined as significant. All statistical analyses were performed in R (R Foundation for Statistical Computing, Vienna, Austria) embedded in the PearlDiver system.

## Results

Between 2010 and 2023, 1,062,597 patients underwent primary elective THA within the dataset. On average, patients who underwent technology-assisted THA had slightly more comorbidities and a shorter length of stay (Table 1). Technology-assisted THA had a higher percentage of cases performed in the inpatient setting (Table 1).

**Table 1:** Patient demographics and characteristics.

Category	Conventional THA		Technology-assisted THA		*P* value
	N	%	N	%	
Total	1,017,887	95.79	44,710	4.21	-
Age (SD)	65.01	10.22	65.00	10.54	.74
CCI (SD)	1.50	1.95	1.52	1.83	<.001^a^
ECI (SD)	4.05	3.23	4.63	3.41	<.001^a^
Length of stay (SD)	3.01	2.30	2.12	1.75	<.001^a^
Sex	-	-	-	-	.45
Male	441,896	43.41	19,491	43.59	-
Female	575,991	56.59	25,219	56.41	-
Payment	-	-	-	-	-
Cash	1597	0.16	122	0.27	<.001^a^
Commercial	648,245	63.69	32,775	73.31	<.001^a^
Government	14,251	1.40	692	1.55	.01^a^
Medicaid	30,380	2.98	15,933	35.64	<.001^a^
Medicare	315,303	30.98	14,200	31.76	<.001^a^
Unknown	8111	0.80	864	1.93	<.001^a^
Inpatient	210,722	20.70	11,119	24.87	<.001^a^

CCI, Charlson Comorbidity Index; ECI, Elixhauser Comorbidity Index; SD, standard deviation.

a Represents significance set at *P* < .05.

**Figure 1:**
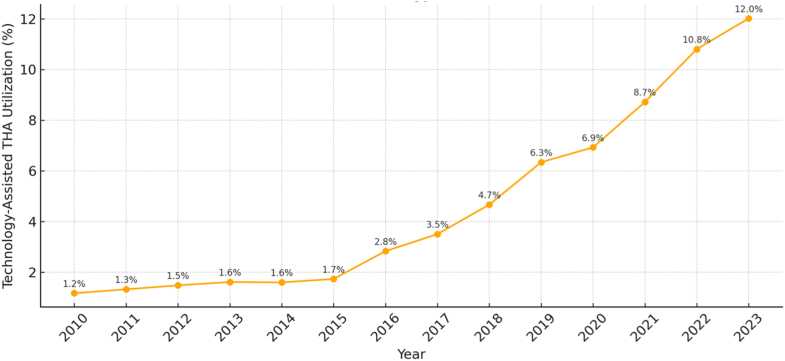
Annual utilization of technology-assisted total hip arthroplasty (THA) in the United States from 2010 to 2023. Percent labels indicate the proportion of all primary elective THAs performed with technology assistance (computer navigation or robotic systems) each year.

**Figure 2:**
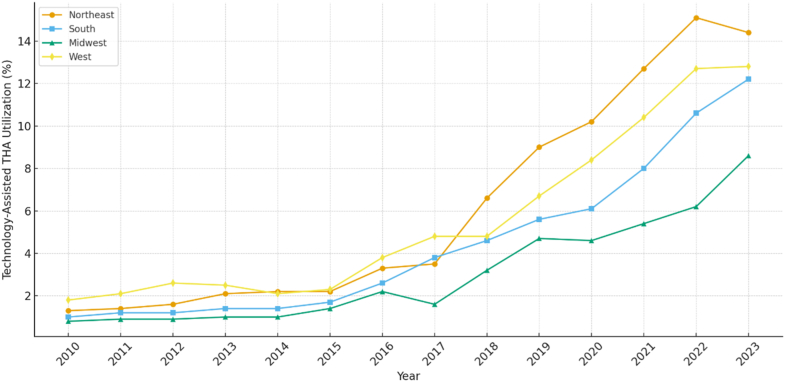
Regional trends in technology-assisted total hip arthroplasty (THA) from 2010 to 2023.

Of all elective THAs performed between 2010 and 2023, 96% were conventional, and 4% were technology-assisted. From 2010 to 2023, the percentage of technology-assisted cases increased annually from 1.2% in 2010 to 12% in 2023, representing an overall increase of 927% (Fig. 1). Utilization of technology-assisted THA varied by region, with the highest overall utilization in the Northeast region and the lowest utilization in the Midwest region (Fig. 2). From 2010 to 2023, use of technology-assisted THA increased by 995% in the Northeast region, 1065% in the Southern region, 955% in the Midwest region, and 600% in the West Coast region (Fig. 2, Appendix 2).

Ninety-day all-cause complications were higher in the conventional THA group (6.26% vs 5.36%; *P* < .001). The rates of medical complications, including pulmonary embolism and pneumonia, and surgical complications, including PJI and dislocation, were higher in the conventional THA group (Table 2). The rate of wound dehiscence was higher in the technology-assisted THA group (Table 2). There were no significant differences in the rate of acute myocardial infarction, mechanical complications, or PPF between the groups. Based on multivariate logistic regression, technology-assisted THA had significantly lower odds of all-cause complications (odds ratio [OR]: 0.77; 95% confidence interval [95% CI]: 0.75-0.80; Table 2). Technology-assisted THA was also associated with significantly decreased odds for dislocation (OR: 0.64; 95% CI: 0.60-0.69), mechanical complications (OR: 0.83; 95% CI: 0.74-0.92), and medical complications (Table 2), and greater odds for wound dehiscence (OR: 1.15; 95% CI: 1.07-1.23).

**Table 2:** Ninety-day complications by method of THA.

Category	Conventional THA		Technology-assisted THA			Technology-assisted vs conventional THA		
	N	%	N	%	*P* value	Adjusted odds ratio	95% confidence interval	*P* value
Total	1,017,887	-	44,710	-	-	-	-	-
Acute myocardial infarction	3537	0.35%	161	0.36%	.69	0.91	0.81-1.03	.13
Pulmonary embolism	7741	0.76%	261	0.58%	<.001^a^	0.67	0.61-0.74	<.001^a^
Pneumonia	11,653	1.14%	435	0.97%	<.001^a^	0.75	0.70-0.81	<.001^a^
Sepsis	5397	0.53%	291	0.65%	<.001^a^	1.00	0.91-1.10	.97
Wound dehiscence	7982	0.78%	435	0.97%	.04^a^	1.15	1.07-1.23	<.001^a^
Mechanical complications	4544	0.45%	179	0.40%	.16	0.83	0.74-0.92	<.001^a^
Periprosthetic joint infection	17,996	1.77%	548	1.23%	.004^a^	0.63	0.59-0.67	<.001^a^
Periprosthetic fracture	7074	0.69%	333	0.74%	.23	0.98	0.90-1.07	.65
Dislocation	13,418	1.32%	406	0.91%	<.001^a^	0.64	0.60-0.69	<.001^a^
All-cause complications	63,700	6.26%	2396	5.36%	<.001^a^	0.77	0.75-0.80	<.001^a^

Adjusted odds ratios (ORs) and 95% confidence intervals were derived from multivariable logistic regression adjusting for age, sex, comorbidity indices, region, and insurance type. *P* values correspond to regression model outputs and were not adjusted for multiple comparisons.

a Represents significance at *P* < .05.

## Discussion

This study sought to determine trends in the utilization of technology-assisted THA and compare 90-day complications across methods for THA. From 2010 to 2023, utilization of technology-assisted THA increased significantly, with annual increases across all regions of the United States. Patients undergoing technology-assisted THA experienced lower odds of 90-day complications, including dislocation and mechanical issues, though with a slightly higher rate of wound dehiscence.

Utilization of technology-assisted THA has increased significantly since 2010, paralleling increasing innovations in THA and growing familiarity with robotic platforms. Our findings align with prior studies reporting similar upward trends. For instance, Hsiue et al. reported a rise in technology-assisted THA from 0.1% in 2005 to 3.0% in 2014, and Korber et al. observed an increase in computer-navigated THA from 0.1% to 1.9% between 2005 and 2018 and an increase in robotic-assisted THA from <0.1% to 2.1% during the same period [7,8]. Our study extends the timeline of analysis and demonstrates that utilization has continued to accelerate beyond the periods previously examined, reaching 12.0% of all primary elective THA in 2023. These results also parallel recent data from the American Joint Replacement Registry, which likewise show increasing adoption of both computer navigation and robotic assistance in THA. The broader adoption of newer robotic platforms—including the Mako Robotic-Arm Assisted Surgery System (Stryker Corporation, Kalamazoo, MI) and the ROSA Hip System (Zimmer Biomet, Warsaw, IN)—may have contributed to the increased utilization observed during the latter portion of the study period [14]. Together, these trends reflect a continued national shift toward technology-assisted approaches in lower extremity arthroplasty.

Regional variation in the utilization of technology-assisted THA was also observed, with the highest overall adoption in the Northeast and the lowest in the Midwest. These findings parallel trends previously reported in technology-assisted total knee arthroplasty (TKA), where the Midwest has consistently demonstrated lower rates of adoption [14,15]. Interestingly, while many prior studies have identified the Western region as a leader in technology utilization, our study found that although the Western region dominated in the early years of the study period, the Northeast region experienced a notable increase in use of technology assistance around 2018, with accelerated adoption that has continued through the end of the study period [7,[14], [15], [16]]. The reasons for this regional shift are not entirely clear, though the introduction and dissemination of newer robotic platforms may have played a role in the increased uptake seen in the Northeast. Early robotic systems such as the ROBODOC Surgical System (originally developed by Integrated Surgical Systems; later commercialized by Curexo/THINK Surgical, Inc., Fremont, CA) were introduced in the 1990s and saw higher early utilization in the Western United States. However, the approval and commercial expansion of newer systems may have facilitated broader national adoption in recent years. Importantly, despite regional differences in overall volume, utilization of technology-assisted THA has increased steadily across all regions of the United States, underscoring an ongoing national trend toward the incorporation of surgical technology in arthroplasty.

While utilization of technology-assisted techniques in THA is steadily increasing, it remains lower than in TKA. Recent data from the American Joint Replacement Registry indicate that robotic assistance is used in approximately 6% of THA cases, compared to nearly 16% in TKA [17]. This discrepancy may, in part, reflect persistent uncertainty regarding the clinical benefit of technology-assisted THA over conventional approaches. In our study, technology assistance was associated with significantly lower odds of 90-day complications, most notably driven by a reduction in dislocation rates. These findings are consistent with previous literature. Namely, Bendich et al. reported a two-thirds reduction in the risk of revision due to dislocation in a single-institution retrospective study of 1770 robotic-assisted THAs, and Piple et al. similarly demonstrated decreased dislocation rates with robotic assistance [11,14]. These reductions are likely attributable to the enhanced ability of robotic systems to optimize acetabular component positioning, leg length, and offset, which are strongly linked to joint stability. In addition to lower dislocation rates, our analysis identified decreased odds of PJI in the technology-assisted group. Although limited data exist on infection risk following technology-assisted THA, Bohl et al. reported a 20% reduction in the risk of PJI with computer navigation compared to conventional THA, and a more recent study observed lower rates of PJI with robotic-assisted THA [11,16]. Despite these benefits, we observed a small but statistically significant increase in wound dehiscence in the technology-assisted group. This finding mirrors observations by Constantinescu et al., who reported higher rates of superficial wound issues in robotic THA potentially related to tracker pin sites [18]. The additional percutaneous incisions and localized soft tissue tension required for registration and tracking may introduce minor soft tissue disruption and contribute to superficial dehiscence. Although the absolute risk increase in our cohort was modest, this suggests that wound-related considerations remain an important aspect of perioperative management when using technology-assisted techniques. Taken together, these findings indicate that technology-assisted THA may offer meaningful reductions in key postoperative complications, particularly dislocation, while highlighting the need for further study to clarify the mechanisms and longer-term implications of wound-related events.

This study has several strengths, including the use of a large, nationally representative administrative claims database, which allowed for the analysis of utilization trends in over one million patients over a 14-year period and across geographic regions. Importantly, this study provides a more contemporary assessment of technology-assisted THA, extending beyond the time frames analyzed in prior literature and incorporating the clinical impact of recently introduced robotic platforms. However, this study should be interpreted in the context of several limitations. First, the use of administrative claims data limits clinical granularity; we were unable to assess surgical approach, implant design, head size, radiographic component positioning, or surgeon volume—operative factors known to influence instability and complication risk, as described by Maratt et al. and Abdel et al [19,20]. Second, although we performed comprehensive multivariable adjustment, the absence of these granular operative variables means that unmeasured confounding and selection bias may still persist and influence 90-day outcomes. Third, the identification of technology-assisted THA relied on billing codes, which may be inconsistently reported across institutions. Lastly, our analysis was limited to 90-day outcomes and does not address long-term survivorship, function, or cost-effectiveness.

## Conclusions

Utilization of technology-assisted THA has increased substantially over the past decade, with notable regional variation and continued growth across all areas of the United States. Technology-assisted THA was associated with lower odds of several short-term complications, particularly dislocation, though a slightly increased risk of wound dehiscence was observed. These findings add to the growing body of literature supporting the potential benefits of surgical technology in arthroplasty and underscore the need for continued evaluation of long-term outcomes and cost-effectiveness.

## Conflicts of interest

I. Bendich received financial or material support from DePuy Synthes Products, Inc., Biocomposites Inc., and Smith & Nephew; all other authors declare no potential conflicts of interest.

For full disclosure statements refer to https://doi.org/10.1016/j.artd.2025.101949.

## CRediT authorship contribution statement

**Amy Y. Zhao:** Writing – original draft, Formal analysis, Data curation. **Alex Gu:** Validation, Supervision, Software, Resources, Methodology. **Gireesh Reddy:** Writing – review & editing, Validation, Conceptualization. **Bryant M. Song:** Writing – review & editing, Validation, Supervision. **Ilya Bendich:** Writing – review & editing, Validation, Project administration, Investigation, Conceptualization. **Andrew Schneider:** Writing – review & editing, Validation, Supervision, Software, Resources, Conceptualization.

## Figures and Tables

**Appendix 1:** Current procedural terminology and international classification of diseases codes.

Variable	CPT/ICD codes
Total hip arthroplasty	CPT-27130, ICD-9-P-8151, ICD-10-P-0SR90J9, ICD-10-P-0SR90JA, ICD-10-P-0SR90JZ, ICD-10-P-0SRB0J9, ICD-10-P-0SRB0JA, ICD-10-P-0SRB0JZ
Technology assistance	ICD-10-P-8E0Y0CZ, ICD-10-P-8E0Y3CZ, ICD-10-P-8E0Y4CZ, ICD-10-P-8E0YXCZ, CPT-S2900, CPT-S29001, ICD-10-P-8E0YXBF, ICD-10-P-8E0YXBG, ICD-10-P-8E0YXBH, ICD-10-P-8E0YXBZ, CPT-0055T, CPT-0054T, CPT-20985
Acute myocardial infarction	ICD-10-D-I2101, ICD-10-D-I2102, ICD-10-D-I2109, ICD-10-D-I2111, ICD-10-D-I2119, ICD-10-D-I2121, ICD-10-D-I2129, ICD-10-D-I213, ICD-10-D-I214, ICD-10-D-I219, ICD-10-D-I21A1, ICD-10-D-I21A9, ICD-10-D-I220, ICD-10-D-I221, ICD-10-D-I222, ICD-10-D-I228, ICD-10-D-I229, ICD-9-D-41001, ICD-9-D-41011, ICD-9-D-41021, ICD-9-D-41031, ICD-9-D-41051, ICD-9-D-41071, ICD-9-D-41081, ICD-9-D-41091
Pulmonary embolism	ICD-10-D-I26, ICD-9-D-4151
Pneumonia	ICD-10-D-A0103, ICD-10-D-A0222, ICD-10-D-A202, ICD-10-D-A212, ICD-10-D-A221, ICD-10-D-A310, ICD-10-D-A3701, ICD-10-D-A3711, ICD-10-D-A430, ICD-10-D-A481, ICD-10-D-B012, ICD-10-D-B052, ICD-10-D-B371, ICD-10-D-B380, ICD-10-D-B381, ICD-10-D-B382, ICD-10-D-B390, ICD-10-D-B391, ICD-10-D-B392, ICD-10-D-B583, ICD-10-D-B59, ICD-10-D-B7781, ICD-10-D-J120, ICD-10-D-J121, ICD-10-D-J122, ICD-10-D-J123, ICD-10-D-J1281, ICD-10-D-J1289, ICD-10-D-J129, ICD-10-D-J13, ICD-10-D-J14, ICD-10-D-J150, ICD-10-D-J151, ICD-10-D-J1520, ICD-10-D-J15211, ICD-10-D-J15212, ICD-10-D-J1529, ICD-10-D-J153, ICD-10-D-J154, ICD-10-D-J155, ICD-10-D-J156, ICD-10-D-J157, ICD-10-D-J158, ICD-10-D-J159, ICD-10-D-J160, ICD-10-D-J168, ICD-10-D-J17, ICD-10-D-J180, ICD-10-D-J181, ICD-10-D-J188, ICD-10-D-J189, ICD-10-D-J851, ICD-10-D-J852, ICD-9-D-1124, ICD-9-D-1140, ICD-9-D-1144, ICD-9-D-1145, ICD-9-D-11505, ICD-9-D-1304, ICD-9-D-1363, ICD-9-D-203, ICD-9-D-212, ICD-9-D-221, ICD-9-D-310, ICD-9-D-4800, ICD-9-D-4801, ICD-9-D-4802, ICD-9-D-4803, ICD-9-D-4808, ICD-9-D-4809, ICD-9-D-481, ICD-9-D-4820, ICD-9-D-4821, ICD-9-D-4822, ICD-9-D-48231, ICD-9-D-48232, ICD-9-D-48240, ICD-9-D-48241, ICD-9-D-48242, ICD-9-D-48249, ICD-9-D-48281, ICD-9-D-48282, ICD-9-D-48283, ICD-9-D-48284, ICD-9-D-4829, ICD-9-D-4830, ICD-9-D-4831, ICD-9-D-4838, ICD-9-D-4847, ICD-9-D-485, ICD-9-D-486, ICD-9-D-5130, ICD-9-D-521, ICD-9-D-551
Sepsis	ICD-10-D-A021, ICD-10-D-A207, ICD-10-D-A227, ICD-10-D-A267, ICD-10-D-A327
	ICD-10-D-A392, ICD-10-D-A393, ICD-10-D-A394, ICD-10-D-A400, ICD-10-D-A401
	ICD-10-D-A403, ICD-10-D-A408, ICD-10-D-A409, ICD-10-D-A4101, ICD-10-D-A4102, ICD-10-D-A411, ICD-10-D-A412
	ICD-10-D-A413, ICD-10-D-A414, ICD-10-D-A4150, ICD-10-D-A4151, ICD-10-D-A4152, ICD-10-D-A4153, ICD-10-D-A4159
	ICD-10-D-A4181, ICD-10-D-A4189
	ICD-10-D-A419, ICD-10-D-A427, ICD-10-D-A5486, ICD-10-D-B007, ICD-10-D-B377
	ICD-10-D-I76, ICD-10-D-P360, ICD-10-D-P3610, ICD-10-D-P3619, ICD-10-D-P362
	ICD-10-D-P3630, ICD-10-D-P3639, ICD-10-D-P364, ICD-10-D-P365, ICD-10-D-P368
	ICD-10-D-P369, ICD-10-D-R6520
	ICD-9-D-1125, ICD-9-D-202, ICD-9-D-223
	ICD-9-D-270, ICD-9-D-271, ICD-9-D-31, ICD-9-D-362, ICD-9-D-380, ICD-9-D-3810, ICD-9-D-3811, ICD-9-D-3812, ICD-9-D-3819, ICD-9-D-383, ICD-9-D-3840, ICD-9-D-3841, ICD-9-D-3842, ICD-9-D-3843, ICD-9-D-3844, ICD-9-D-3849, ICD-9-D-388, ICD-9-D-389, ICD-9-D-449, ICD-9-D-545, ICD-9-D-77181, ICD-9-D-9889, ICD-9-D-99592
Wound dehiscence	ICD-10-D-T8130XA, ICD-10-D-T8130XD, ICD-10-D-T8130XS, ICD-10-D-T8131XA, ICD-10-D-T8131XD, ICD-10-D-T8131XS
	ICD-10-D-T8132XA, ICD-10-D-T8132XD
	ICD-10-D-T8132XS, ICD-10-D-T8133XA
	ICD-10-D-T8133XD, ICD-10-D-T8133XS, ICD-9-D-99830, ICD-9-D-99831, ICD-9-D-99832, ICD-9-D-99833
Mechanical complications	ICD-10-D-T84030A, ICD-10-D-T84031A, ICD-10-D-T84039A, ICD-9-D-99641, ICD-10-D-T84090A, ICD-10-D-T84091A, ICD-10-D-T84098A, ICD-10-D-T84099A, ICD-9-D-99647, ICD-8-D-99649
Periprosthetic joint infection	ICD-9-D-99666, ICD-10-D-T845, ICD-10-D-T8450, ICD-10-D-T8450XA, ICD-10-D-T8451, ICD-10-D-T8451XA, ICD-10-D-T8452, ICD-10-D-T8452XA, ICD-10-D-T8459, ICD-10-D-T8459XA
Periprosthetic fracture	ICD-10-D-M9701XA, ICD-10-D-M9702XA, ICD-10-D-M970, ICD-10-D-M978, ICD-10-D-M979, ICD-10-D-T84040A, ICD-10-D-T84041A, ICD-9-D-99644
Dislocation	ICD-10-D-T84020A, ICD-10-D-T84021A, ICD-10-D-T84028A, ICD-10-D-T84029A, ICD-9-D-99642

**Appendix 2:**
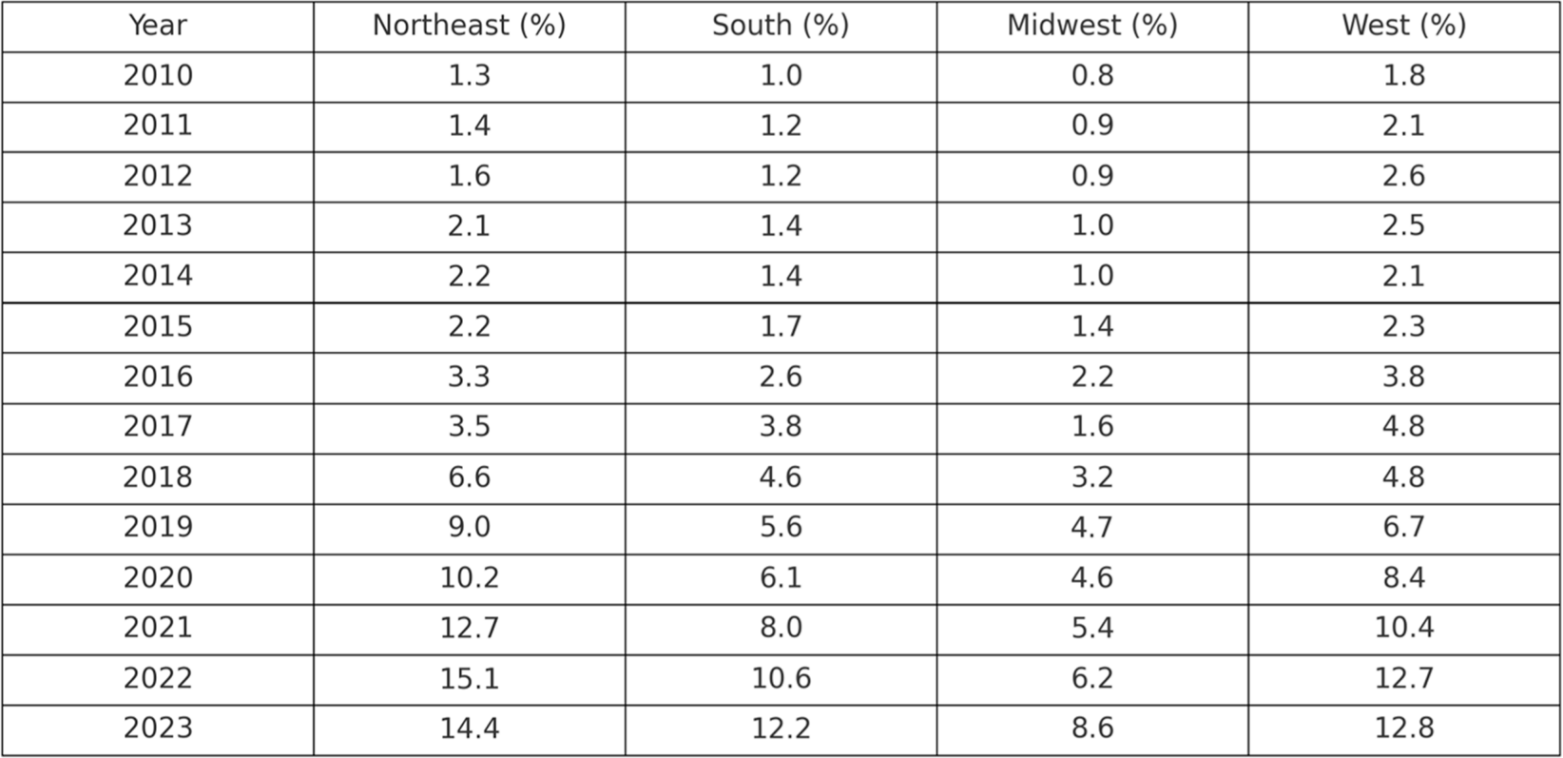
Percent utilization of technology-assisted total hip arthroplasty by U.S. Census Region, 2010-2023.

## Appendix A Supplementary data

Conflict of Interest Statement for Bendich

Conflict of Interest Statement for Gu

Conflict of Interest Statement for Reddy

Conflict of Interest Statement for Schneider

Conflict of Interest Statement for Song

Conflict of Interest Statement for Zhao
