# Supplementary material for: Technology-assisted Total Hip Arthroplasty: A Contemporary Analysis of Regional Trends, National Trends, and 90-day Outcomes in a Nationwide Cohort
Source: Arthroplast Today. 2026 Jan 31;38:101949. doi: 10.1016/j.artd.2025.101949 (PMC12877833; doi:10.1016/j.artd.2025.101949)
Supplement: Conflict of Interest Statement for Song [file mmc5.docx]

# INDIVIDUAL CONFLICT OF INTEREST STATEMENT

***American Association of Hip and Knee Surgeons***

(Adopted from the American Academy of Orthopaedic Surgeons disclosure statement)

The following form **must be filled out completely and submitted by each author (example, 6 authors, 6 forms).**

**All items require a response. If there is no relevant disclosure for a given item, enter "*None*.”**

**Manuscript Title: Technology-Assisted Total Hip Arthroplasty: A Contemporary Analysis of Regional Trends, National Trends, and 90-day Outcomes in a Nationwide Cohort**

1. Royalties from a company or supplier (The following conflicts were disclosed)

None

2. Speakers bureau/paid presentations for a company or supplier (The following conflicts were disclosed)

None

3A. Paid employee for a company or supplier (The following conflicts were disclosed)

None

3B. Paid consultant for a company or supplier (The following conflicts were disclosed)

None

3C. Unpaid consultants for a company or supplier (The following conflicts were disclosed)

None

4. Stock or stock options in a company or supplier (The following conflicts were disclosed)

None

5. Research support from a company or supplier as a Principal Investigator (The following conflicts were disclosed)

None

6. Other financial or material support from a company or supplier (The following conflicts were disclosed)

None

7. Royalties, financial or material support from publishers (The following conflicts were disclosed)

None

8. Medical/Orthopaedic publications editorial/governing board (The following conflicts were disclosed)

None

9. Board member/committee appointments for a society (The following conflicts were disclosed)

None

**Each author must sign AND print or type his/her name, date and submit a separate form**

In addition, one BLINDED Conflict of Interest form (no author names used) should be submitted per manuscript with all author disclosures.

Bryant Song Bryant Song 3/31/2025

Author Name (Print or Type) Author Signature Date
